# Luteolin Mitigates Photoaging Caused by UVA-Induced Fibroblast Senescence by Modulating Oxidative Stress Pathways

**DOI:** 10.3390/ijms26051809

**Published:** 2025-02-20

**Authors:** Yu Yan, Haiting Huang, Tongshan Su, Wenyi Huang, Xinyu Wu, Xianxian Chen, Sen Ye, Jun Zhong, Chun Li, Yu Li

**Affiliations:** 1 School of Nursing, Guangzhou University of Chinese Medicine, Guangzhou 510405, China; yanyu@gzucm.edu.cn (Y.Y.); 20211110331@stu.gzucm.edu.cn (H.H.); 13076599552@163.com (T.S.); huangwy1212@163.com (W.H.); xinw6611@163.com (X.W.); chenxianxian2000@outlook.com (X.C.); 2 Nursing Interdisciplinary Platform, Guangzhou University of Chinese Medicine, Guangzhou 510405, China; 3 Research Center of Integrative Medicine, School of Basic Medical Sciences, Guangzhou University of Chinese Medicine, Guangzhou 510405, China; yesen@gzucm.edu.cn (S.Y.); jade_zhong@foxmail.com (J.Z.); 4 School of Basic Medical Sciences, Guangzhou University of Chinese Medicine, Guangzhou 510405, China

**Keywords:** luteolin, UVA-induced photoaging, fibroblast senescence, oxidative stress, antioxidant effect, collagen degradation

## Abstract

As a polyphenolic plant flavone, luteolin (Lut) is widely found in many medicinal plants, flowers, and vegetables. Although Lut has been shown to have the effect of preventing and treating skin photoaging, its role in preventing photoaging specifically induced by ultraviolet A (UVA) radiation remains underreported. In vivo, BALB/c mice were used as models for skin photoaging models and treated with Lut. Additionally, NIH-3T3 fibroblasts were utilized in vitro to further investigate whether Lut exerts its anti-photoaging effects by enhancing fibroblast vitality and function. Several biochemical assays (CCK-8, catalase, superoxide dismutase, malondialdehyde, dichloro-dihydro-fluorescein diacetate, quantitative real-time-PCR, gene expression patterns) and histochemical (histological staining, immunofluorescent staining, SA-β-Gal experiments, western blotting analysis) were conducted. The findings demonstrate that the Lut pretreatment could enhance the vitality and function of fibroblasts in both in vitro and in vivo experiments and inhibit UVA-induced collagen degradation, thereby improving the skin’s resistance to photoaging. We confirmed that the Lut pretreatment inhibited the expression of UVA-induced senescent factors P21, P16, and pro-inflammatory senescence-associated secretory phenotype (SASP) factors. Additionally, Lut exhibited potent antioxidant effects during UVA exposure. Bioinformatics and network pharmacology analyses revealed that Lut’s anti-photoaging effects may be mediated through the regulation of oxidative stress-related pathways and anti-aging genes. Upon utilizing inhibitors and agonists of oxidative stress, we further confirmed that Lut prevents UVA-induced fibroblast senescence by suppressing oxidative stress, and ultimately protects the skin from photoaging damage. These findings indicate that lutein mitigates photoaging caused by UVA-induced fibroblast senescence through the modulation of oxidative stress pathways.

## 1. Introduction

Skin photoaging is chronic damage to the skin caused by ultraviolet (UV) rays which manifests itself as dryness, wrinkles, roughness, irregular pigmentation, and loss of skin elasticity, etc. In severe cases, it can result in deep plough furrows, milia, open acne, actinic purpura, and the thickening of the epidermis and dermis, and can even lead to the development of cutaneous malignant tumors [1,2,3,4]. In recent years, skin photoaging has emerged as a key area of interest in the fields of dermatology and medical aesthetic care [5,6,7]. The treatments for skin photoaging include drugs, surgery, and stem cells [8,9]. However, these treatments often involve high costs, adverse drug reactions, or postoperative complications. Given the unavoidable exposure to ultraviolet radiation in daily life, the prevention of skin photoaging is crucial.

Similar to naturally aging skin, photoaged skin exhibits a decrease in collagen and elastin, leading to wrinkle formation, loss of elasticity, hyperpigmentation, and dryness of the skin [10]. However, photoaged skin exposed to ultraviolet radiation (UV) also exhibits a thickening of the epidermal layer and hyperkeratosis [8,11]. Aging is a major risk factor for the development of chronic diseases, and skin aging occurs with age or exposure to environmental factors such as UV. This process can propagate the aging phenotype from the skin to other tissues and organs through SASPs, thereby contributing to overall systemic aging [12]. Mitochondrial dysfunction is considered one of the determinants of aging, leading to the accumulation of reactive oxygen species (ROS) which, in turn, promotes cellular senescence [13].

Luteolin (3′, 4′, 5′, 7-tetrahydroxyflavones, Lut) is a flavone with various pharmacological properties such as anti-inflammatory, antibacterial, antioxidant, and antitumor activities [14]. It comes from a wide range of sources and is found in many medicinal plants, fruit, flowers, vegetables, spices, and purgative and detoxifying drugs such as grape, carrot, orange, amaranth, etc. Moreover, Lut is relatively easy to isolate and obtain. At micromolar concentrations, Lut demonstrates a strong anti-inflammatory activity [15,16], with its most significant function being its potent antioxidant capacity [17]. Lut is considered a safer antioxidant substance. Recent studies have shown that Lut exhibits photoprotective effects against ultraviolet B (UVB)-induced photodamage in zebrafish, primarily through DNA protection, antioxidant, and anti-inflammatory effects [18]. However, ultraviolet B primarily affects the epidermal layer and the superficial dermis, whereas ultraviolet A (UVA), which accounts for 90–95% of the total UV spectrum, is considered to be the main cause of skin photoaging [4]. Therefore, the photoaging damage caused by UVA to the skin is more significant and lasting, making the prevention of UVA-induced skin photoaging particularly significant. Studies have confirmed that flavonoids play a significant role in preventing UVA-induced skin photoaging [19]. As one of the flavones, the potential protective effect of Lut against UVA-induced skin photoaging and its mechanism have not been fully elucidated. Therefore, in order to explore the application value of Lut in preventing skin photoaging, this study aims to systematically explore the protective effect of Lut on UVA-induced fibroblast senescence and its mechanism in a mammal model.

## 2. Results

### 2.1. Luteolin Can Reduce UVA-Induced Skin Photoaging In Vivo

**Figure 1:**
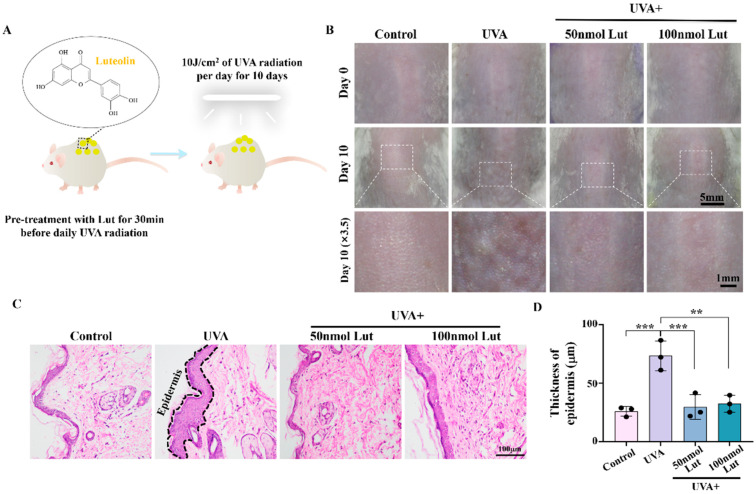
Assessment of the protective effect of the luteolin pretreatment on UVA-induced skin photoaging in vivo. (**A**) Schematic diagram of the modeling of the drug pretreatment photoaging animal model. (**B**) Representative images of the dorsal skin of BALB/c mice after 10 consecutive days of UVA radiation. (**C**) Representative H&E-stained transverse sections of dorsal skin for each group on day 10. (**D**) Bar chart showing the thickness of the epidermis on day 10. ** *p* < 0.01, *** *p* < 0.001 compared with the UVA group.

The dorsal skin of BALB/c mice was irradiated with 10 J/cm^2^ UVA radiation for 10 consecutive days. Moreover, mice were pretreated with Lut prior to the daily UVA exposure (Figure 1A). The skin morphology of UVA-irradiated mice exhibited increased roughness, wrinkling, and dryness compared to the control group, suggesting skin damage associated with photoaging. Lut pretreatment significantly alleviated photoaging following UVA radiation compared to the UVA group. The 100 nmol Lut + UVA group demonstrated the most notable improvement in skin photoaging (Figure 1B). Additionally, H&E staining of the dorsal skin tissue on day 10 showed that the Lut pretreatment could inhibit the UVA-induced thickening of the skin epidermis, suggesting that Lut could effectively prevent the skin photoaging damage caused by UVA (Figure 1C,D).

### 2.2. Luteolin Can Inhibit UVA-Induced Collagen Degradation In Vitro and In Vivo

**Figure 2:**
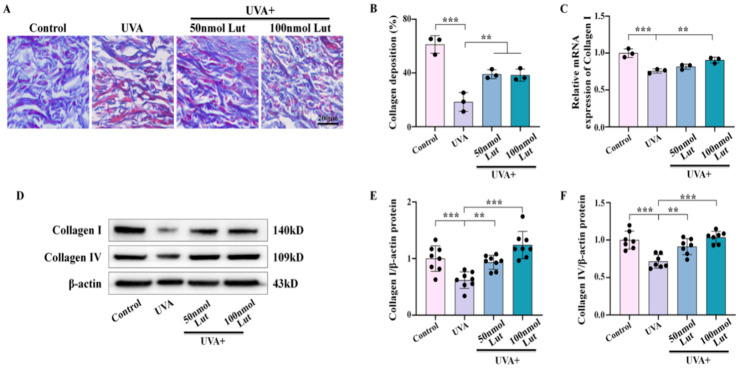
Assessment of luteolin in preventing UVA-induced collagen degradation in vivo. (**A**) Representative Masson-stained transverse sections of dorsal skin for each group on day 10. (**B**) Bar chart showing the collagen deposition on day 10. (**C**) Bar chart showing the mRNA expression levels of Collagen I. (**D**–**F**) Western blot images and bar charts showing the total protein of Collagen I and Collagen IV in each group on day 10. ** *p* < 0.01, *** *p* < 0.001 compared with the UVA group.

Photoaging can cause many histological changes in the skin, including decreased collagen synthesis, increased collagen fragmentation, and a subsequent reduction in the overall collagen content [20,21]. To confirm that Lut has a preventive effect on UVA-induced skin photoaging, we used Masson staining to stain the dorsal skin of mice that were continuously irradiated with UVA for 10 days. The results showed that the Lut pretreatment can effectively prevent the degradation of dermal collagen during photoaging (Figure 2A,B). Collagen I is the most abundant component of the dermal extracellular matrix, while Collagen IV is an important reticular fiber component in the dermis. The decrease in the content of both collagen types will lead to a decrease in collagen production [22]. We first used q-PCR to confirm that the Lut pretreatment can reduce the downregulation of Collagen I expression caused by UVA (Figure 2C). Furthermore, western blot experiments demonstrated that the decrease in Collagen I and Collagen IV protein levels induced by UVA was reversed following the Lut pretreatment (Figure 2D–F). The above results suggest that Lut can prevent the decrease in collagen content in vivo to promote its anti-photoaging effect.

**Figure 3:**
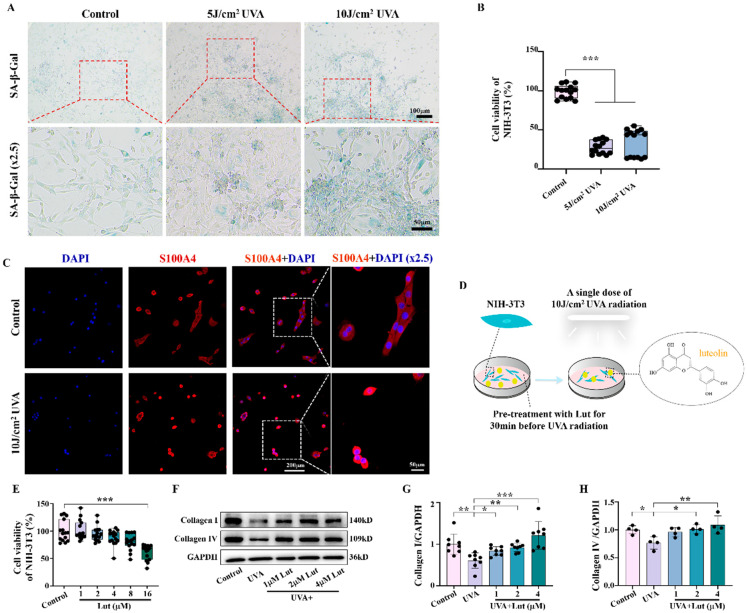
Assessment of luteolin in preventing the UVA-induced decrease in fibroblast activity and collagen degradation in vitro. (**A**) Representative images of SA-β-Gal staining on the control, continuous 3 days of 5 J/cm^2^ UVA radiation, and single 10 J/cm^2^ UVA radiation groups. (**B**) Cell viability of NIH-3T3 on the control, continuous 3 days of 5 J/cm^2^ UVA radiation, and single 10 J/cm^2^ UVA radiation groups. (**C**) Representative immunofluorescence staining for S100A4 in the control and 10 J/cm^2^ UVA groups. (**D**) Schematic diagram of the cell model for drug prevention of UVA-induced photoaging. (**E**) Cell viability of NIH-3T3 after treatment with various concentrations of Lut. (**F**–**H**) Western blot images and bar charts showing the total protein of Collagen I and Collagen IV in each group. * *p* < 0.05, ** *p* < 0.01, *** *p* < 0.001 compared with the Control or UVA group.

As we all know, fibroblasts are the main cell components that produce and maintain the extracellular matrix of the dermis. They can synthesize and secrete collagen and elastin and generate collagen fibers, reticular fibers, and elastic fibers [23]. In this study, we used NIH-3T3 to conduct further experiments in vitro to verify whether Lut plays an anti-photoaging role by promoting the vitality and function of fibroblasts. First, the SA-β-Gal assay and cell activity assay showed that a single dose of 10 J/cm^2^ UVA radiation induced a more pronounced trend of cellular senescence in vitro compared to a single dose of 5 J/cm^2^ UVA radiation, and was, therefore, more suitable for constructing in vitro photoaging models (Figure 3A,B). After using S100A4 to mark the morphology of the fibroblasts, we found that a single dose of 10 J/cm^2^ UVA radiation can cause morphological changes such as cell collapse, atrophy, and cytoplasmic vacuolation, indicating that UVA radiation can indeed cause fibroblast senescence and decreased cell activity (Figure 3C). Next, we used Lut to treat UVA-radiated NIH-3T3 for 30 min to assess the protective effect of Lut against UVA-induced cell senescence (Figure 3D). We first exposed the cells to different concentrations of Lut and assessed cell viability to determine the optimal treatment concentration (Figure 3E). Western blot results showed that UVA radiation inhibited the secretion of collagen by cells. However, this inhibitory effect was significantly alleviated after treatment with different concentrations of Lut (Figure 3F–H).

### 2.3. Luteolin Pretreatment Can Prevent UVA-Induced Photoaging by Enhancing the Anti-Aging Capacity of Fibroblasts

**Figure 4:**
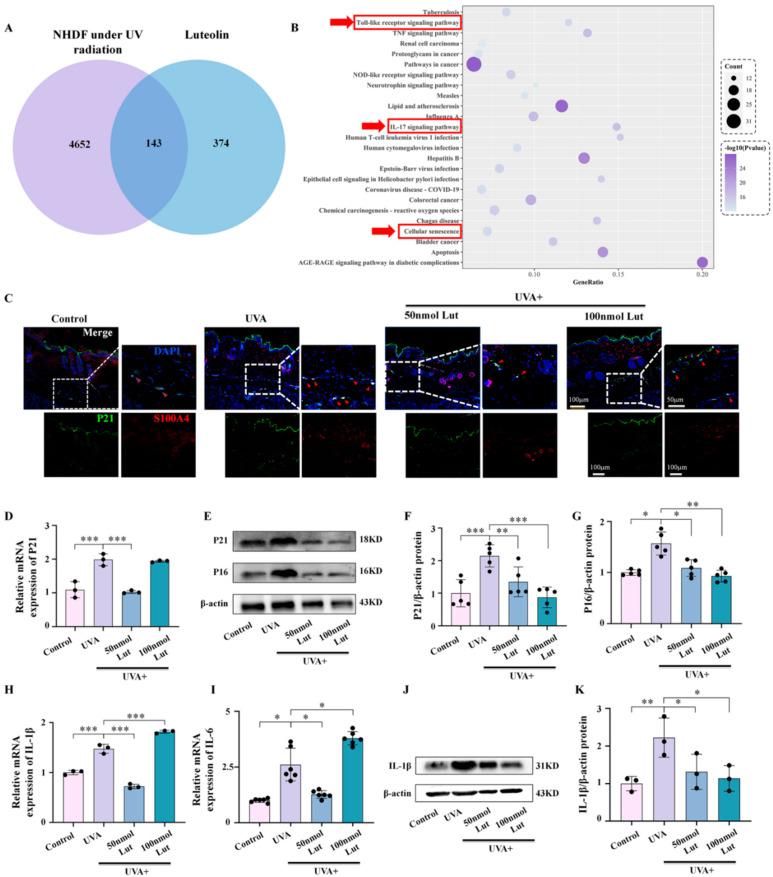
Assessment of luteolin in preventing UVA-induced cell senescence in vivo. (**A**) Cross-gene map of the transcriptome genes affected by UV on human dermal fibroblasts and targets of Lut. (**B**) R language mapping of the top 25 KEGG pathways enriched with common target genes in (**A**). (**C**) Representative immunofluorescence double staining images of P21 and S100A4 in the dorsal skin of mice on day 10 in each group. (**D**) Bar chart showing the mRNA expression levels of P21 in the dorsal skin of mice on day 10 in each group. (**E**–**G**) Western blot images and bar charts showing the total protein of P21 and P16 in the dorsal skin of mice on day 10 in each group. (**H**,**I**) Bar chart showing the mRNA expression levels of IL-1β and IL-6 in the dorsal skin of mice on day 10 in each group. (**J**,**K**) Western blot images and bar charts showing the total protein of IL-1β in the dorsal skin of mice on day 10 in each group. * *p* < 0.05, ** *p* < 0.01, *** *p* < 0.001 compared with the UVA group.

Based on the above experimental results, we found that Lut may play an anti-photoaging role by mediating the function of fibroblasts. To further clarify the mechanism, we first used the GEO database to retrieve 607 differentially expressed genes of human dermal fibroblasts after ultraviolet radiation. We then utilized TCMSP, TCD, PharmMapper, and Swisstarget Prediction 19 to retrieve 517 target genes of Lut. After the intersection of these two groups of gene sets, we identified 143 common target genes between Lut and the photoaged human dermal fibroblasts which accounted for 28% of the target genes of Lut. This result suggests that Lut may effectively target fibroblast damage caused by photoaging (Figure 4A). After enrichment analysis, we found that these target genes are related to signaling pathways such as the toll-like receptor signaling pathway, the IL-17 signaling pathway, and cellular senescence. These results suggest that Lut may mitigate UVA-induced fibroblast senescence and inflammatory responses (Figure 4B). The results of double immunofluorescence labeling of S100A4 and P21 showed that the Lut pretreatment could reduce the increase in the senescence of dermal fibroblasts caused by UVA (Figure 4C). At the same time, q-PCR and western blot were used to assess the expression of the aging-related genes P21 and P16 (Figure 4D–G) and the inflammatory markers IL-1β and IL-6 (Figure 4H–K), respectively. Their levels were both significantly downregulated after the Lut pretreatment. These analyses confirmed the protective effect of Lut pretreatment on the senescence of fibroblasts.

### 2.4. Luteolin Effectively Protects Fibroblasts from Photoaging Caused by UVA In Vitro

**Figure 5:**
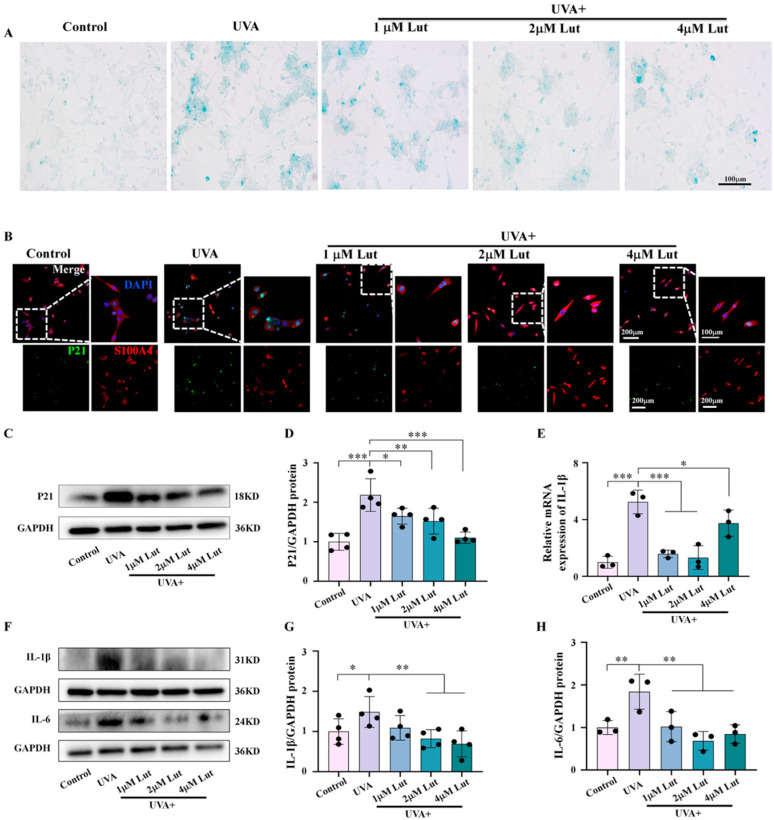
Assessment of luteolin in preventing UVA-induced cell senescence in vitro. (**A**) Representative images of SA-β-Gal staining of NIH-3T3 in each group. (**B**) Representative immunofluorescence double staining images of P21 and S100A4 of NIH-3T3 in each group. (**C**,**D**) Western blot images and bar charts showing the total protein of P21 of NIH-3T3 in each group. (**E**) Bar chart showing the mRNA expression levels of IL-1β of NIH-3T3 in each group. (**F**–**H**) Western blot images and bar charts showing the total protein of IL-1β and IL-6. Bar chart showing the mRNA expression levels of IL-1β of NIH-3T3 in each group * *p* < 0.05, ** *p* < 0.01, *** *p* < 0.001 compared with the UVA group.

To further clarify the protective effect of Lut on the senescence of fibroblasts, we conducted additional in vitro verification experiments using NIH-3T3 cells. The results of the SA-β-Gal test showed that UVA-induced cell senescence could be effectively inhibited by an increase in the concentration of the Lut pretreatment (Figure 5A). The results of the immunofluorescence staining and western blot showed that the Lut pretreatment inhibited the increase in the number of P21-positive cells and protein expression caused by UVA (Figure 5B–D). Simultaneously, the results of western blot and q-PCR showed that the increase in the expression levels of the inflammatory markers IL-1β and IL-6 of NIH-3T3 caused by UVA was significantly downregulated after the Lut pretreatment (Figure 5E–H). All of the above results can confirm the preventive and protective effects of Lut on UVA-induced fibroblast senescence in vitro.

### 2.5. Luteolin Exhibits Strong Antioxidant Effects Against UVA-Induced Photoaging

**Figure 6:**
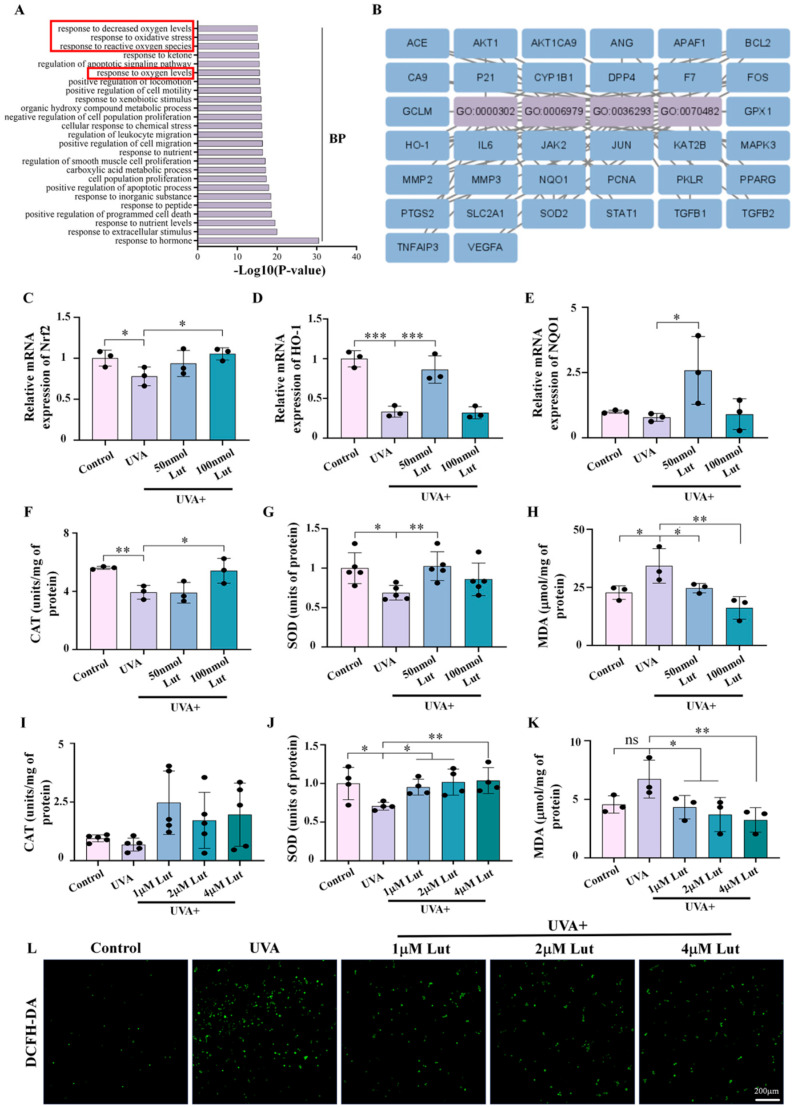
Assessment of the strong antioxidant effects of luteolin in in vivo and in vitro photoaging models. (**A**) Top 25 GO biological process maps of the cross genes in Figure 4A. (**B**) Genes corresponding to the oxidative stress biological process are enriched in Figure 6A. (**C**–**E**) Bar charts showing the mRNA expression levels of Nrf2, HO-1, and NQO1 in the dorsal skin of mice on day 10 in each group. (**F**–**H**) Bar charts showing CAT, SOD, and MDA expression levels in the dorsal skin of mice on day 10 in each group. (**I**–**K**) Bar charts showing CAT, SOD, and MDA expression levels in NIH-3T3 in each group. (**L**) Representative images of DCFH-DA staining of NIH-3T3 in each group. ns *p >* 0.05, * *p* < 0.05, ** *p* < 0.01, *** *p* < 0.001 compared with the UVA group.

GO analysis of common target genes between Lut and photoaged human dermal fibroblasts provides strong data support for our subsequent research of specific mechanisms. The results of the GO analysis showed that the top 25 enriched entries included several categories related to the response to the oxidative status, such as response to decreased oxygen levels, response to oxidative stress, response to reactive oxygen species, and response to oxygen levels. These results suggest that oxidative stress may be involved in Lut’s prevention and protection against UVA-induced photoaging (Figure 6A,B). Next, we quantified the expression levels of several common target genes in the oxidative stress-related pathways and found that Lut could reverse the decrease in the expression of Nrf2, HO-1, and NQO1 induced by UVA (Figure 6C–E). Further analysis of the concentrations of the antioxidant enzymes CAT and SOD, as well as the oxidative stress marker MDA, in photoaged skin tissues and NIH-3T3 revealed that UVA could cause a decrease in the content of antioxidant enzymes and an increase in the level of oxidative stress in both skin tissues and fibroblasts. However, the adverse effects of oxidative stress caused by UVA were attenuated after the pretreatment with Lut (Figure 6F–K). At the same time, after using DCFH-DA staining to assess the level of ROS in NIH-3T3, we were able to confirm the antioxidant role of Lut in the process of photoaging induced by UVA (Figure 6L).

### 2.6. Oxidative Stress Is an Important Mediating Mechanism of Luteolin in the Process of Photoaging Caused by UVA

**Figure 7:**
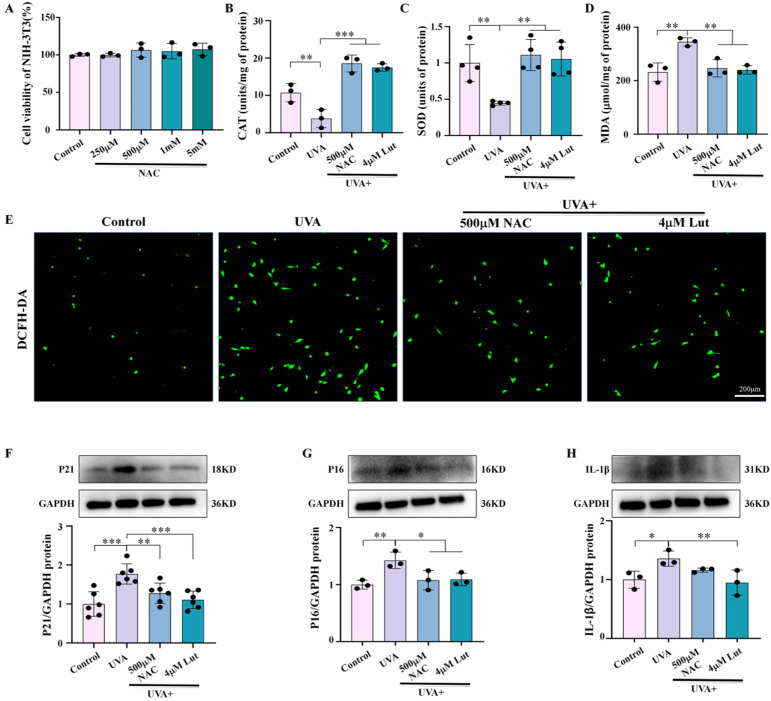
Assessment of the stronger antioxidant and anti-aging effects of luteolin compared with the classic antioxidant NAC under UVA radiation in vitro. (**A**) Cell viability of NIH-3T3 after treatment with various concentrations of NAC. (**B**–**D**) Bar charts showing CAT, SOD, and MDA expression levels in NIH-3T3 in each group. (**E**) Representative images of DCFH-DA staining of NIH-3T3 in each group. (**F**–**H**) Western blot images and bar charts showing the total protein of P21, P16, and IL-1β of NIH-3T3 in each group. * *p* < 0.05, ** *p* < 0.01, *** *p* < 0.001 compared with the UVA group.

To clarify the central role of oxidative stress in UVA-induced photoaging and confirm it as the key mechanism by which Lut prevents cellular senescence, we pretreated NIH-3T3 cells with the classic antioxidant NAC and Lut. The results demonstrate Lut’s superior antioxidant and anti-photoaging potential under UVA radiation. The optimal NAC concentration was determined by a cell activity test (Figure 7A). The results obtained by detecting oxidative stress-related indicators showed that Lut had a better antioxidant effect than NAC under UVA conditions (Figure 7B–E). In addition, compared with NAC treatment groups, Lut could more significantly downregulate the increased expression levels of P21, P16, and IL-β in NIH-3T3 caused by UVA, indicating that Lut has a better anti-photoaging potential than NAC under UVA radiation (Figure 7F–H).

**Figure 8:**
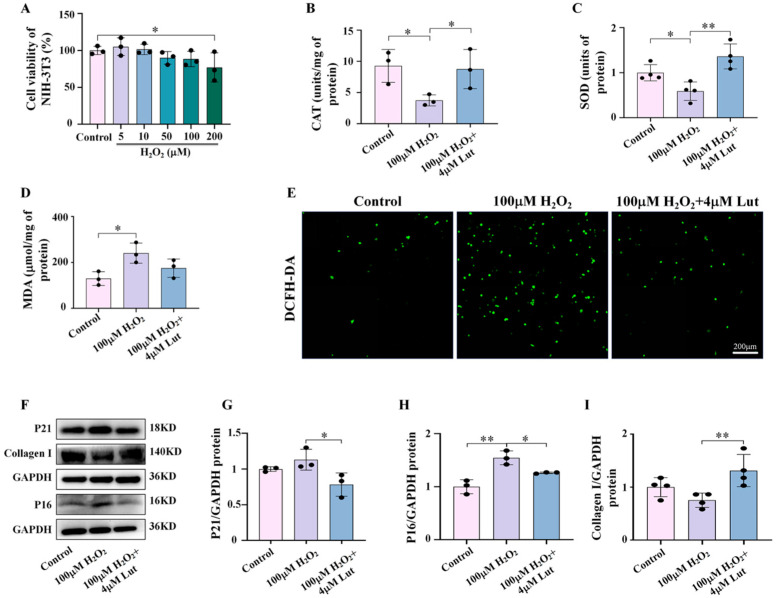
Assessing the dominant role of oxidative stress in anti-aging processes in vitro. (**A**) Cell viability of NIH-3T3 after treatment with various concentrations of H_2_O_2_. (**B**–**D**) Bar charts showing CAT, SOD, and MDA expression levels in NIH-3T3 in each group. (**E**) Representative images of DCFH-DA staining of NIH-3T3 in each group. (**F**–**I**) Western blot images and bar charts showing the total protein of P21, P16, and Collagen I of NIH-3T3 in each group. * *p* < 0.05, ** *p* < 0.01 compared with the H_2_O_2_ group.

Finally, we used H_2_O_2_ (a commonly used oxidative stress activator) to treat NIH-3T3 with Lut. The experiment was conducted with 100 M H_2_O_2_, which was determined to have no toxic side effects on cells through cell activity tests (Figure 8A). The measurement of CAT and SOD concentrations revealed that Lut could reverse the decrease in antioxidant enzyme levels caused by H_2_O_2_ (Figure 8B,C). Simultaneously, the increase in MDA concentration and DCFH-DA fluorescence intensity caused by the H_2_O_2_ treatment was also mitigated by treatment with Lut (Figure 8D,E), suggesting that Lut does have significant antioxidant effects. Furthermore, using western blot to analyze the expression levels of the aging-related factors P21 and P16 and Collagen I, we found that H_2_O_2_ induced aging in NIH-3T3 cells and further inhibited Collagen I secretion. However, Lut treatment can mitigate these negative effects of oxidative stress on cells (Figure 8F–I).

## 3. Discussion

UVA radiation is the main pathogenic factor of skin photoaging and can accelerate the aging process of the skin and even cause cancer by inducing mechanisms such as DNA damage, oxidative stress, and cell senescence [24]. Lut is a natural compound that has been found to have multiple pharmacological activities, but its role and potential mechanism in preventing UVA-induced skin photoaging have not been fully clarified. In this study, we first demonstrated that Lut can effectively alleviate UVA-induced skin photoaging in vivo. By subjecting BALB/c mice to 10 J/cm^2^ of UVA radiation for 10 consecutive days, we observed increased wrinkles and epidermal thickening in the dorsal skin of the mice, successfully establishing a photoaging model. These changes are consistent with known signs of photoaging, including structural changes in the dermis and epidermis of the skin. They are primarily due to a compensatory thickening of the stratum corneum following UVA exposure which helps to block part of the ultraviolet rays. Moreover, ultraviolet rays activate tyrosinase to protect cells, resulting in a decrease in the dermal collagen content and reduced skin elasticity. After the Lut pretreatment, these UVA-induced changes were significantly reduced, indicating that Lut has a protective effect against UVA-induced photoaging damage (Figure 1). Previous studies have shown that flavonoid natural compounds can protect against UV-induced skin damage to a certain extent [25,26,27]. However, most of these studies have primarily focused on UVB. In this study, we demonstrate the therapeutic efficacy of Lut against UVA-induced skin photoaging, highlighting its potential as a more comprehensive photoprotective agent.

Collagen is a key component of the skin structure and function, and its reduction in content is an important marker of photoaging. Our results show that the Lut pretreatment significantly inhibited UVA-induced collagen degradation. Previous studies have confirmed that, in photoaging, overexpression of MMPs leads to aggravated collagen degradation and damaged skin structure [28]. Lut extracted from plants can inhibit the expression or activity of MMPs, which is one of the important anti-aging strategies [29]. Collagen I and collagen IV are the main components of the dermal fibrous structure and the reticular fibrous structure of the basement membrane, respectively. In this study, we confirmed that Lut significantly inhibits UVA-induced degradation of collagen I and further demonstrated its protective effect on collagen IV (Figure 2). This finding expands our understanding of the potential mechanism of Lut in the prevention and treatment of photoaging, indicating that Lut can comprehensively enhance the skin’s ability to resist UVA by maintaining the content of multiple key collagens in the dermis.

Fibroblasts are the primary cells that maintain the dermal matrix. When their function is impaired, it will lead to reduced collagen synthesis, which, in turn, accelerates skin photoaging. Through our study on NIH-3T3 fibroblasts, we found that Lut can significantly reduce UVA-induced fibroblast senescence. We further confirmed the anti-photoaging activity of Lut using Res, a compound that has been shown to have significant anti-photoaging effects, as a positive control (Supplementary Figure S1). Furthermore, we confirmed that Lut could restore the collagen synthesis ability of fibroblasts, including the promotion of collagen I and collagen IV. These findings suggest that fibroblasts are the main target cells for Lut to exert its anti-photoaging effect. Therefore, Lut may protect the skin from photoaging damage by improving fibroblast function (Figure 3). This is consistent with an emerging hypothesis on skin aging proposed by Meinhard Wlaschek et al. which suggests that senescent fibroblasts have an intrinsic ability to escape apoptosis and elimination by the adaptive immune system, thus playing a major role in the irreversible skin aging process [30].

The relationship between cellular senescence and inflammatory responses is close and complex. They interact in multiple biological and pathophysiological processes, forming a self-reinforcing feedback loop. Cellular senescence is accompanied by SASP. One of its characteristics is that senescent cells secrete a large number of inflammatory factors, cytokines, chemokines, and proteases. SASP triggers and maintains inflammatory responses at local and systemic levels through these secreted substances, and this inflammatory environment induces more cells to enter a state of senescence. We explored the potential mechanism of Lut in anti-photoaging through bioinformatics methods. A total of 607 differentially expressed genes in human dermal fibroblasts after ultraviolet radiation exposure were screened out through the GEO database. By integrating these data with the Lut target gene database analysis, we identified 143 common target genes between Lut and photoaged human dermal fibroblasts. These genes are involved in multiple key signaling pathways closely related to inflammation and cellular senescence, such as the toll-like receptor signaling pathway, the IL-17 signaling pathway, and the cellular senescence pathway. At the same time, we further confirmed, through in vivo and in vitro experiments, that the Lut pretreatment can significantly reduce the expression levels of the UVA-induced fibroblast senescence markers P21 and P16, as well as the expression of the inflammatory factors IL-1β and IL-6 (Figure 4 and Figure 5).

Further investigation into the potential mechanism of Lut’s anti-photoaging effects revealed that Lut significantly mitigates UVA-induced oxidative stress by regulating the expression of key antioxidant genes, including Nrf2, HO-1, and NQO1, and by increasing the levels of antioxidant enzymes such as CAT and SOD (Figure 6). This is consistent with the antioxidant capacity of Lut reported in previous studies. Lut is involved in protecting skin cells from UVB radiation-induced cellular senescence through the SIRT3/ROS/MAPK axis [31]. In addition, NAC is a widely used antioxidant, and its antioxidant properties have been confirmed in multiple studies. Through comparative experiments with the classic antioxidant NAC, we found that Lut exhibits better antioxidant and anti-photoaging effects. Our study shows that Lut is significantly more effective than classic antioxidants in dealing with skin photoaging induced by UVA (Figure 7). Similarly, our study confirmed the central role of oxidative stress in Lut’s anti-photoaging function through H_2_O_2_ activation experiments (Figure 8). This finding has important clinical implications as it provides strong evidence for Lut as a more effective natural anti-photoaging agent.

## 4. Materials and Methods

### 4.1. Animal Experiment

BALB/c mice (5–6 weeks old, weighing 18–22 g) were purchased from Guangdong Pharmachem Biotechnology Co., Ltd. (Foshan, China) (License No: SYXK (GD) 2018-0182). All mice were housed in SPF-grade conditions at a temperature of 25 ± 2 °C, with a 12/12 h light–dark cycle and free access to water. Subsequently, the mice were randomly divided into four groups (*n* = 6): control group, UVA group (the mice were exposed to 10 J/cm^2^ UVA for 10 days), 50 nmol Lut + UVA group, and 100 nmol Lut + UVA group. Specifically, we first dissolved Lut powder with DMSO (D12345, ThermoFisher, Waltham, MA, USA) and then further diluted it to 50 nmol or 100 nmol with ultrapure water, ensuring that the dilution factor was greater than 1000 times to avoid the potential toxicity of DMSO. Before exposing the mice’s back skin to 10 J/cm^2^ UVA every day, we pretreated the skin with 200 μL of 50 nmol or 100 nmol Lut for 30 min. To ensure that Lut was evenly distributed on the skin, we thoroughly vortexed Lut and cleaned the mouse back skin to remove surface dirt and oil before each treatment. Then, Lut was evenly applied to the designated area of the skin to ensure a full distribution and penetration. This procedure was repeated once daily and, after 10 consecutive days of treatment, the skin samples were photographed and collected. A UVA lamp (TL-K40W10R, Philips, Amsterdam, The Netherlands) was used to irradiate the mouse skin, and the radiation energy was calculated using a UV energy meter (UV-150, Shenzhen Speedre Technology, Shenzhen, China). All animal experiment procedures were approved by the Animal Experiment Center of Guangzhou University of Chinese Medicine (Animal Ethics No: 20230522009, Approval Date: 22 May 2023).

### 4.2. Tissue Preparation and Histological Staining

After the mice skin tissue was collected and fixed in 4% paraformaldehyde (BL539A, Biosharp, Hefei, China) overnight, the tissue was dehydrated in 50%, 70%, 80%, 90%, 95%, and 100% ethanol in sequence and then transparentized with xylene. The tissue block was immersed in paraffin at 60 °C and placed in an embedding box for paraffin embedding. The embedded tissue was cut into 4–6 μm with a paraffin slicer (RM2235, Leica, Wetzlan, Germany) for subsequent staining.

Paraffin sections were stained with H&E [32] and Masson [33] staining following previously established protocols. Specifically, after dewaxing and hydration, skin tissue sections were stained with hematoxylin (FD7443, FUDE Biology, Hangzhou, China) and eosin (FD7443, FUDE Biology, China) for H&E staining. Masson staining was performed with the Masson staining kit (C0189S, Beyotime, Shanghai, China). All sections were then mounted with neutral gum (G6590G, Biotopped, Beijing, China) and imaged using a Leica DM57 light microscope (DM57, Leica, Wetzlan, Germany).

### 4.3. Cell Culture and Treatment

NIH-3T3 cells (FH0983, Fuheng Biology, Shanghai, China) were cultured using Dulbecco’s modified eagle’s medium (DMEM, C11995500BT, Gibco, Grand Island, NY, USA), containing 10% fetal bovine serum (16410071, Gibco, Grand Island, NY, USA) and 1% streptomycin and penicillin (15140122, Gibco, USA), in an incubator at 37 °C with 5% CO_2_. The medium was changed every 2 days. When the cells reached the appropriate density, they were treated with or without different concentrations of Lut, resveratrol (Res), N-acetylcysteine (NAC), and/or H_2_O_2_, according to experimental needs.

### 4.4. Immunofluorescent Staining

Tissue sections were deparaffinized in xylene for 1 h and then hydrated by immersion in anhydrous ethanol, 95% ethanol, 80% ethanol, 70% ethanol, and ultrapure water for 10 min each. The membrane was repaired using sodium citrate antigen repair solution (AR0024, Boster, Wuhan, China) at 60 °C overnight. Subsequently, the membrane was washed with PBS, then permeabilized with 0.5% Triton X-100 for 30 min, and blocked with 10% goat serum for 30 min. It was then titrated with the primary antibodies: P21 (1:200, 28248-1-AP, Proteintech, Wuhan, China) and S100A4 (1:200, 66489-1-Ig, Proteintech, Wuhan, China) for overnight incubation at 4 °C. After washing, PBST (0.1% Tween-20) was used. Fluorescent secondary antibodies, either Alexa Fluor 488 (1:500, AB150077, Abcam, Cambridge, MA, USA) or Alexa Fluor 647 (1:500, AB150115, Abcam, USA), were applied and incubated at room temperature in the dark for 1 h. DAPI (P0131-25ml, Beyotime, Shanghai, China) was added for 5 min. The samples were observed and imaged using a confocal microscope (ZEISS, Oberkochen, Germany).

NIH-3T3 cells were planted on cell crawls (5 × 10^3^ cells/cm^2^) and pretreated with or without different drugs 30 min before 10 J/cm^2^ UVA irradiation. The cells were divided into a control group, a UVA group, a 10 μM Res + UVA group, a 1 μM Lut + UVA group, a 2 μM Lut + UVA group, a 4 μM Lut + UVA group, a 500 mM NAC + UVA group, a 100 mM H_2_O_2_ group, and a 100 mM H_2_O_2_ + 4 μM Lut group. The cells were fixed with 4% paraformaldehyde for 30 min and then permeabilized for 30 min. The following steps were consistent with the tissue immunofluorescence as described above.

### 4.5. SA-β-Gal Experiments

Staining experiments were performed according to the instructions of the kit (C0602, Beyotime, Shanghai, China). Cells were cultured in six-well dishes (8 × 10^4^ cells/cm^2^) and pretreated with or without different concentration of Lut 30 min before UVA irradiation. The cells were divided into a control group, a 5 J/cm^2^ UVA group (cells received UVA radiation at 5 J/cm^2^ per day for 3 days), a 10 J/cm^2^ UVA group (cells received a single dose of UVA radiation at 10 J/cm^2^), a 1 μM Lut + UVA group (10 J/cm^2^ UVA irradiation), a 2 μM Lut + UVA group (10 J/cm^2^ UVA irradiation), and a 4 μM Lut + UVA group (10 J/cm^2^ UVA irradiation). The cells were fixed for 30 min and then incubated with a staining solution at 37 °C in an oven overnight. Subsequently, sections were sealed with anti-fluorescence quencher (P0126-5ml, Beyotime, Shanghai, China) for 12 h and then imaged using a Leica DM57 optical microscope.

### 4.6. CCK-8 Experiment

NIH-3T3 cells were inoculated in 96-well plates (1.5 × 10^3^ cells/cm^2^), and were divided into the following groups: a control group, a 5 J/cm^2^ UVA group, a 10 J/cm^2^ UVA group, different concentrations of Lut (1 μM, 2 μM, 4 μM, 8 μM, 16 μM) groups, different concentrations of oxidative stress inhibitor N-acetylcysteine (NAC) (S1623, Selleck, Houston, TX, USA) (250 μM, 500 μM, 1 mM, 5 mM) groups, and different concentrations of oxidative stress agonist hydrogen peroxide (H_2_O_2_) (AAPR555-CA100, Pythonbio, Guangzhou, China) (5 μM, 10 μM, 50 μM, 100 μM, 200 μM) groups. The cells were cultured for 24 h and then experiments were carried out using the CCK-8 kit (C0038, Beyotime, Shanghai, China). Following a 30 min incubation at 37 °C with 5% CO_2_, the optical density (OD) values at 450 nm were measured using a Bio-Rad 450 microplate reader (Bio-Rad, Hercules, CA, USA).

### 4.7. Catalase (CAT) Activity Assay

Total protein of skin tissues or NIH-3T3 cells was extracted using PBS, and experiments were performed according to the instructions of the kit (S0051, Beyotime, Shanghai, China). Briefly, tissues and cells were grinded using PBS to collect protein fluids. The tissues were divided into a control group, a UVA group, a 50 nmol Lut + UVA group, and a 100 nmol Lut + UVA group. The cells were divided into a control group, a UVA group (the cells were irradiated with 10 J/cm^2^ UVA), a 1 μM Lut + UVA group, a 2 μM Lut + UVA group, a 4 μM Lut + UVA group, a 500 μM NAC + UVA group (500 μM NAC was used to pretreat the cells for 30 min before the 10 J/cm^2^ UVA irradiation), a 100 μM H_2_O_2_ group (cells were incubated with 100 μM H_2_O_2_ at 37 °C for 30 min), and a 100μM H_2_O_2_ + 4μM Lut group (cells were incubated with 100 μM H_2_O_2_ + 4 μM Lut at 37 °C for 30 min). After that, the CAT working solution was added and incubated at room temperature for 30 min, and then the absorbance value was measured at 520 nm using a Bio-Rad 450 microplate reader.

### 4.8. Superoxide Dismutase (SOD) Activity Assay

Total protein of skin tissues or NIH-3T3 cells was extracted using PBS, and experiments were performed according to the instructions of the kit (S0109, Beyotime, Shanghai, China). Briefly, tissues and cells were grinded using PBS to collect protein fluids. The tissues were divided into a control group, a UVA group, a 50 nmol Lut + UVA group, and a 100 nmol Lut + UVA group. The cells were divided into a control group, a UVA group, a 1 μM Lut + UVA group, a 2 μM Lut + UVA group, a 4 μM Lut + UVA group, a 500 μM NAC + UVA group, a 100 μM H_2_O_2_ group, and a 100 μM H_2_O_2_ + 4 μM Lut group. Subsequently, the SOD assay working solution was added and incubated at 37 °C for 30 min, and then the absorbance value was measured at 560 nm using a Bio-Rad 450 microplate reader.

### 4.9. Malondialdehyde (MDA) Activity Assay

Total protein of skin tissues or NIH-3T3 cells was extracted using PBS, and experiments were performed according to instructions of the kit (S0131S, Beyotime, Shanghai, China). Briefly, the tissues and NIH-3T3 cells were grinded using PBS to collect protein fluids. The tissues were divided into a control group, a UVA group, a 50 nmol Lut + UVA group, and a 100 nmol Lut + UVA group. The cells were divided into a control group, a UVA group, a 1 μM Lut + UVA group, a 2 μM Lut + UVA group, a 4 μM Lut + UVA group, a 500 μM NAC + UVA group, a 100 μM H_2_O_2_ group, and a 100 μM H_2_O_2_ + 4 μM Lut group. After that, the MDA assay working solution was added and heated for 15 min. The samples were then allowed to return to room temperature and centrifuged at 1000× *g* for 10 min, and then the absorbance value was measured at 532 nm using a Bio-Rad 450 microplate reader.

### 4.10. Dichloro-Dihydro-Fluorescein Diacetate (DCFH-DA) Assay

NIH-3T3 cells were planted in 24-well plates (5 × 10^3^ cells/cm^2^) and the experiments were performed according to the instructions of the kit (S0033S, Beyotime, Shanghai, China). The cells were divided into a control group, a UVA group, a 4 μM Lut + UVA group, a 500 μM NAC + UVA group, a 100 μM H_2_O_2_ group, and a 100 μM H_2_O_2_ + 4 μM Lut group. Briefly, the cells were treated with a DCFH-DA working solution and incubated for 20 min at 37 °C in a cell culture incubator, protected from light, and then imaged using an Olympus IX51 inverted fluorescent microscope (IX51, Olympus Corporation, Tokyo, Japan).

### 4.11. Quantitative Real-Time-PCR (RT-qPCR)

Mouse skin tissues were divided into a control group, a UVA group, a 50 nmol Lut + UVA group, and a 100 nmol Lut + UVA group. The cells were divided into a control group, a UVA group, a 1 μM Lut + UVA group, a 2 μM Lut + UVA group, a 4 μM Lut + UVA group, a 500 μM NAC + UVA group, a 100 μM H_2_O_2_ group, and a 100 μM H_2_O_2_ + 4 μM Lut group. In brief, total RNA from skin tissues and cells was extracted using the TRIZOL kit (15596018, Invitrogen, Shanghai, China), cDNA was synthesized using a reverse transcription kit (A230-10, GenStar, Beijing, China), and q-PCR was performed using q-PCR Mix (A301-10, GenStar, China). The sequences are listed in Supplementary Table S1.

### 4.12. Western Blotting Analysis

Skin tissues or cellular proteins were lysed using RIPA lysate (FD009, FUDE Biology, Hangzhou, China). Total protein concentration was determined using the BCA method (FD2001, FUDE Biology, Hangzhou, China). All proteins were then subjected to SDS-PAGE and then transferred to preactivated polyvinylidene difluoride membranes (PVDF, Bio-Rad, Hercules, CA, USA). After blocking with 5% skimmed milk (9999S, Cell Signaling Technology, Danvers, MA, USA), the primary antibodies i.e., collagen I (1:1000, 66761-1-Ig, Proteintech, Wuhan, China), collagen IV (1:1000, SD83-03, HUABIO, Hangzhou, China), P21 (1:1000, 10355-1-AP, Proteintech, Wuhan, China), P16 (1:1000, sc-1661, Santa Cruz Biotechnology, USA), IL-1β (1:1000, 12703S, Cell Signaling Technology, Danvers, MA, USA), and IL-6 (1:1000, 12912S, Cell Signaling Technology, Danvers, MA, USA), were incubated overnight at 4 °C in a refrigerator. The secondary antibodies, rabbit (1:1000, 7074S, Cell Signaling Technology, Danvers, MA, USA) or mouse (1:2000, 7076S, Cell Signaling Technology, Danvers, MA, USA), were used every other day to bind to the primary antibodies. Images were developed using a luminescent solution (WBKLS0500, Millipore, Shanghai, China) and captured with a fully automated chemiluminescent fluorescence image analysis system (5200Multi, Tanon, Shanghai, China).

### 4.13. Gene Enrichment Analysis Based on Bioinformatics and Network Pharmacology

The GEO database (https://www.ncbi.nlm.nih.gov/geo/, accessed on 20 August 2023) was searched by entering the keyword “photoaging” to obtain 4795 genes from the dataset GSE11900918 (transcriptomic expression analysis of the effects of ultraviolet radiation on normal human dermal fibroblasts). A total of 517 Lut targets were obtained from TCMSP, TCD, PharmMapper, and Swisstarget Prediction19. Gene IDs were converted to gene names using the uniprot website (https://www.uniprot.org/, accessed on 20 August 2023). Gene intersections were obtained using the VENNY2.1 website (https://bioinfogp.cnb.csic.es/tools/venny/, accessed on 20 August 2023). The Metascape website (https://metascape.org/gp/index.html#/main/step1, accessed on 20 August 2023) was used to obtain KEGG-enriched pathways, and KEGG pathway bubble plots were generated using R version 4.2.0. Intersecting genes were analyzed using the GO analysis results from the Metascape website (https://metascape.org/gp/index.html#/main/step1), and GO bioprocess maps were drawn using GraphPad Prism 8.3.

### 4.14. Data Analysis

The SPSS 25.0 software and GraphPad Prism 8.3 were used for data analysis and statistical graphing. The experiments were repeated at least three times. One-way analyses of variance or Kruskal–Wallis tests were used to compare multiple groups of data, and the results are expressed as the mean ± standard deviation or median. * *p* < 0.05 was considered statistically significant, ** *p* < 0.01 and *** *p* < 0.001 were considered highly statistically significant.

## 5. Conclusions

**Figure 9:**
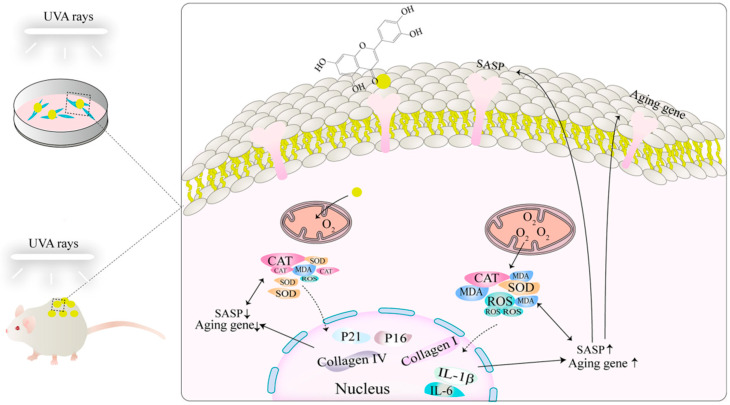
Schematic diagram of the mechanism of luteolin in preventing UVA-induced photoaging.

In summary, this study innovatively targets the problem of UVA radiation-induced skin photoaging and comprehensively evaluates the protective and therapeutic potential of the natural compound Lut. Different from previous studies that focused on UVB-induced photoaging or cellular levels, this study reveals the significant role of Lut in alleviating UVA-induced skin photoaging through in vivo and in vitro experiments. Its potential mechanisms were explored through multiple pathways such as inhibiting collagen degradation, improving fibroblast function, and regulating the expression of antioxidant genes and aging-related genes (Figure 9). Furthermore, using bioinformatics and network pharmacology analysis, we systematically revealed the multi-faceted molecular mechanism of Lut in preventing and treating UVA-induced skin photoaging. Upon comparing it with the classic antioxidant NAC, we confirmed the therapeutic potential of Lut in antioxidation and anti-photoaging. Further in-depth and extensive research should be conducted in the future to prove the safety and efficacy of Lut. In addition, interdisciplinary and multi-level investigations are essential to effectively transform the results of basic research into clinical application strategies.

## Data Availability

The GEO database (https://www.ncbi.nlm.nih.gov/geo/, accessed on 20 August 2023). The uniprot website (https://www.uniprot.org/). The VENNY2.1 website (https://bioinfogp.cnb.csic.es/tools/venny/). The Metascape website (https://metascape.org/gp/index.html#/main/step1).

## Supplementary Materials

The following supporting information can be downloaded at: https://www.mdpi.com/article/10.3390/ijms26051809/s1.
