# Supplementary material for: Luteolin Mitigates Photoaging Caused by UVA-Induced Fibroblast Senescence by Modulating Oxidative Stress Pathways
Source: Int J Mol Sci. 2025 Feb 20;26(5):1809. doi: 10.3390/ijms26051809 (PMC11899068; doi:10.3390/ijms26051809)
Supplement: Supplementary file 1 [file ijms-26-01809-s001.zip › ijms-3417254-supplementary.pdf]

Supplementary Figure:

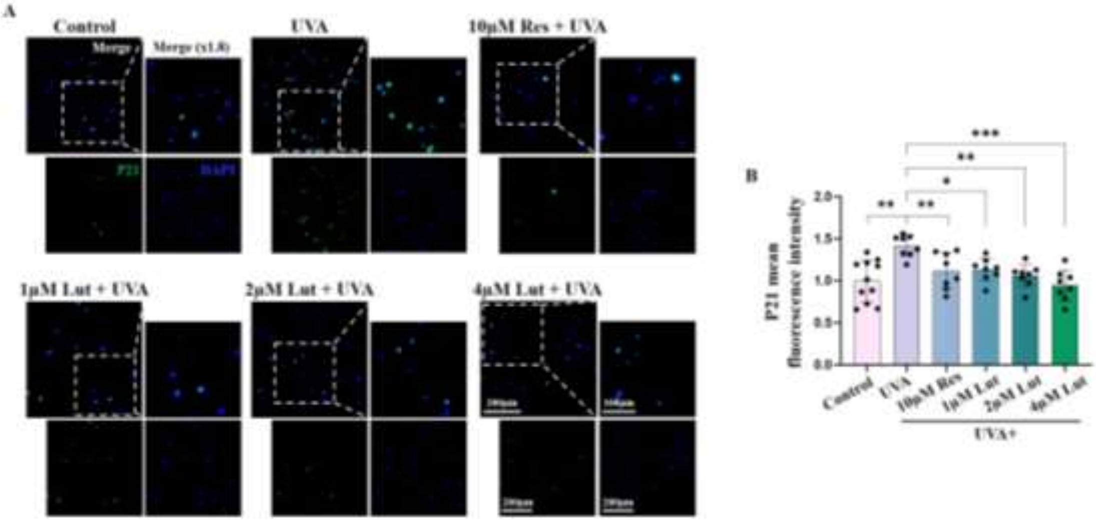

Supplementary Figure S1. Assessment of resveratrol and luteolin in preventing UVA-induced cell senescence in vitro. (A) Representative immunofluorescence double staining images of P21 in NIH-3T3 in each group. （B）Bar chart showing mean fluorescence intensity of P21 in each group. *P<0.05, **P<0.01 compared with the UVA group.

Supplementary Table S1 Primer sequences for q-PCR used in the study

Primers	Forward	Reverse
Collagen Ⅰ	GACAGGCGAACAAGGTGACAGAG	CAGGAGAACCAGGAGAACCAGGAG
P21	CCTTGTCGCTGTCTTGCACTCTG	GCTGGTCTGCCTCCGTTTTCG
IL-1β	TCGCAGCAGCACATCAACAAGAG	AGGTCCACGGGAAAGACACAGG
IL-6	CTTCTTGGGACTGATGCTGGTGAC	TCTGTTGGGAGTGGTATCCTCTGTG
Nrf2	TGCCACCGCCAGGACTACAG	GCGTGCTCAGAAACCTCCTTCC
HO-1	ACCGCCTTCCTGCTCAACATTG	CTCTGACGAAGTGACGCCATCTG
NQO1	GCGAGAAGAGCCCTGATTGTACTG	AGCCTCTACAGCAGCCTCCTTC
